# Neurohormonal Effects of Intravenous Dopamine in Patients with Acute Heart Failure

**DOI:** 10.3390/jcm13195667

**Published:** 2024-09-24

**Authors:** Christos Kourek, Andrew Xanthopoulos, Grigorios Giamouzis, Charalambos Parisis, Alexandros Briasoulis, Dimitrios E. Magouliotis, Filippos Triposkiadis, John Skoularigis

**Affiliations:** 1 Department of Cardiology, 417 Army Share Fund Hospital of Athens (NIMTS), 11521 Athens, Greece; chris.kourek.92@gmail.com; 2 Department of Cardiology, University Hospital of Larissa, 41110 Larissa, Greece; grgiamouzis@gmail.com; 3 404 General Military Hospital, 41222 Larissa, Greece; charalambosparisis@yahoo.gr; 4 Department of Clinical Therapeutics, Alexandra Hospital, National and Kapodistrian University of Athens, 15772 Athens, Greece; alexbriasoulis@gmail.com; 5 Unit of Quality Improvement, Department of Cardiothoracic Surgery, University of Thessaly, 41110 Biopolis, Greece; dmagouliotis@gmail.com; 6 School of Medicine, European University Cyprus, 2404 Nicosia, Cyprus; ftriposkiadis@gmail.com

**Keywords:** dopamine, acute heart failure, furosemide, neurohormonal, creatinine, renal function, clinical outcomes

## Abstract

**Background/Objectives**: Many clinical trials have shown beneficial effects of low-dose dopamine on renal function, diuresis and symptom relief, or cardiac function in hospitalized patients with acute decompensated heart failure (HF). The aim is to assess the neurohormonal effects and the effects on clinical outcomes of the addition of low-dose dopamine in furosemide treatment in patients hospitalized for acute decompensated HF. **Methods**: A total of 62 patients hospitalized for acute decompensation of HF, were randomly allocated to one of the following three groups: i. LDF (low-dose furosemide), ii. HDF (high-dose furosemide) and, iii. LDFD (low-dose furosemide and dopamine). Primary outcomes of the present analysis were biochemical and neurohormonal indices (i.e., urea, creatinine, hemoglobin, electrolytes, natriuretic peptides, troponin, renin, angiotensin, aldosterone, adrenaline, noradrenaline). Secondary endpoints included clinical outcomes (i.e., length of stay, in-hospital mortality, 2-month mortality and rehospitalization, and 1-year mortality and rehospitalization). **Results**: Urea and creatinine levels were similar for each day among the three groups (*p* > 0.05). The amount of urine was similar among the three groups per measurement at 2, 4, 6 and at 8 h (*p* > 0.05). Biochemical and neurohormonal indices as well as clinical outcomes did not differ among patients receiving different doses of furosemide, nor in patients receiving furosemide in combination with dopamine (*p* > 0.05). **Conclusions**: Although the addition of low-dose dopamine to intravenous furosemide was considered to have some theoretical advantages in maintaining renal function, no significant differences in neurohormonal effects and clinical outcomes were observed in patients hospitalized for acute decompensation of HF.

## 1. Introduction

Worsening renal function (WRF), defined as an increase ≥ 0.3 mg/dL (26.5 mmol/L) in the serum creatinine level compared with the value on admission [1], has been proven to be associated with prolonged hospital stay, higher in-hospital mortality, increased likelihood of readmission, and increased mortality after discharge in hospitalized patients for acute decompensated heart failure (HF) [2,3]. Furosemide is considered as a first-line therapy in acute decompensated HF leading to a decrease in ventricular filling pressures and improvement in symptoms [4]. The diuretic effect of intravenous (IV) administration of furosemide starts within 30 min and its peak is at 1.5 h [4]. WRF is approximately 25% to 40% in acute decompensated HF patients under IV furosemide [5].

Dopamine is a catecholamine which may selectively activate dopamine receptors and promote splanchnic, coronary and renal vasodilation at low doses (≤3 μg/kg/min) [6,7]. Dopamine administration at moderate doses (3–5 μg/kg per min) augments cardiac contraction and chronotropy through stimulation of the cardiac beta-1 receptors and increases the pulmonary capillary wedge pressure [7,8] while at higher doses (>5 lg/kg per min) it stimulates alpha-1 receptors producing vasoconstriction, leading, thus, to a significantly elevated afterload that may prove detrimental for patients with acute HF and systolic dysfunction [7,8]. Additionally, dopamine can activate the renal dopamine receptor (D1), dilate the renal arteries, and increase urine output [9]. The combination of low-dose furosemide and low-dose dopamine has been shown to be equally effective as high-dose furosemide, but associated with improved renal function profile and potassium homeostasis in patients hospitalized with acute decompensated HF [10]. Moreover, many clinical trials have shown beneficial effects of low-dose dopamine on renal function, diuresis and symptom relief [11,12], or cardiac function in hospitalized patients with acute HF [12]. However, whether the addition of dopamine in furosemide therapy in acute decompensated HF has beneficial neurohormonal effects is unknown.

The aim of the study was to compare the neurohormonal effects of low-dose furosemide and dopamine with continuous intravenous administration versus low- or high-dose furosemide only, in patients hospitalized for acute decompensated HF, as well as to evaluate fluctuations of plasma dopamine levels and their correlation with clinical outcomes.

## 2. Materials and Methods

### 2.1. Study Design

The present work was a secondary analysis of the multicenter DAD-HF II trial [13] and included data from patients hospitalized for acute decompensation of HF (2009–2012) from our center (Larissa University Hospital, Greece). The study was conducted in accordance with the ethical guidelines of the 1975 Declaration of Helsinki and approved by the Administration Board and the Ethics Committee of University Hospital of Larissa, Greece [ClinicalTrials.gov Identifier: NCT00937092 (http://clinicaltrials.gov/ct2/show/NCT00937092, accessed on 5 September 2024)]. Each patient signed an informed consent form for the procedure and study participation. The study was not sponsored by industry support and was funded locally.

### 2.2. Patients and Procedure

Patient were eligible to participate if they were >18 years old, were admitted for dyspnea on minimal exertion or rest dyspnea with an oxygen saturation < 90% on admission arterial blood gas, and had one or more of the following: (a) signs of congestion (third heart sound or pulmonary rales > 1/3 or lower extremity/sacral edema > 1+ on examination), (b) interstitial congestion or pleural effusion on chest X-ray, and (c) serum B-type natriuretic peptide levels > 400 pg/mL or NT-proBNP > 1500 pg/mL. Key exclusion criteria were as follows: (a) serum creatinine > 200 μmol/L or GFR < 30 mL min^−1^ 1.73 m^−2^, (b) systolic blood pressure < 90 mmHg, (c) severe valvular disease, (d) known adverse reactions to furosemide or dopamine, (e) complex congenital heart disease, (f) anticipated need for IV contrast use, (g) suspected or confirmed acute coronary syndrome on admission, and (h) a scheduled cardiac surgery within 6 months [10,13].

All patients were randomized in one of the following 3 groups: i. Group 1 (LDF, low-dose furosemide) with continuous intravenous furosemide of 5 mg per hour for a total of 8 h, ii. Group 2 (HDF, high-dose furosemide) with continuous intravenous furosemide of 20 mg per hour for a total of 8 h, and iii. Group 3 (LDFD, low-dose furosemide and dopamine) with continuous intravenous administration of 5 mg furosemide per hour in combination with continuous intravenous administration of 5 μg/kg/min dopamine for a total of 8 h. All patients were administered an initial loading dose of 40 mg of intravenous furosemide. 

Blood samples were taken to determine biochemical markers [hemoglobin, electrolytes, brain natriuretic peptide (BNP) and renal function indices] upon emergency department admission, at 8 h, at 24 h and thereafter, once daily. At the same time, plasma levels of dopamine, renin, angiotensin, aldosterone, adrenaline and noradrenaline were determined upon emergency department admission and after 8 h. After patients fasted overnight (for at least 12 h), venous blood samples were drawn from an antecubital vein in the morning between 8:00 and 9:00 a.m. in the supine position at rest for at least 20 min, after the venipuncture. The samples were immediately centrifuged and stored at −30 °C for further analysis. Total plasma adrenaline, noradrenaline and dopamine levels were measured using human RIA (Radioimmunoassay) diagnostic kits (3-CAT KIPL 1600), produced by DiaSource Europe SA (Louvain-la-Neuve, Belgium). The kit has a sensitivity of 7.5 pg/mL for adrenalin, 37.5 pg/mL for noradrenaline and 20 pg/mL for dopamine, a less-than-1% cross-reaction with various biogenic amines, their derivatives and structural related compounds, while the intra- and inter-assay coefficients of variation range between 4.6–13.9% and 5.6–6.1% for adrenaline, 4.0–4.6% and 6.1–10.1% for noradrenaline and 6.1–8.1% and 9.0–13.4% for dopamine, respectively. Plasma rennin levels were measured using human IRMA (Immunoradiometric assay) diagnostic kits (KIP1531), produced by DiaSource Europe SA (Belgium). The kit has a sensitivity of 0.78 pg/mL for renin, and the prorenin cross-reactivity was found to be 0.3%, while the intra- and inter-assay coefficients of variation ranged between 3.0–8.5% and 4–11%, respectively. Total serum aldosterone levels were measured using human RIA diagnostic kits (RVR-CW-100), produced by ZenTech SA (Liège, Belgium). The kit has a sensitivity of 1.4 pg/mL sensitivity, and a 17.2% cross-reaction with 3β-5α tetrahydroaldosterone (the cross-reaction with other compounds is less than 1%), while the intra- and inter-assay coefficients of variation ranged between 5.3 and 13.7% and 6.2 and 18.6%, respectively. Plasma rennin activity (angiotensin I) was determined using human RIA diagnostic kits (RVR-EX-125), produced by ZenTech SA (Belgium). The kit has a sensitivity of 0.033 ng/mL for angiotensin I, the cross-reactivity with interfering compounds is minimal (less than 0.32%) and the intra- and inter-assay coefficients of variation range between 3.39–6.04% and 3.82–5.15%, respectively. Moreover, all RIA and IRMA kits are calibrated against valid international standards. The radiotracer used in all kits is 125 Iodine (I-125, half-life T1/2 60 days, 35.5 keV gamma radiation, 27–32 keV X-rays, no beta radiation). All sample assays were performed duplicated, included in the same run for each biological parameter. If the difference between duplicate results of a sample was more than 5%, the sample assay was repeated, while the in-run coefficients of variation were 4.1% for adrenaline, 4.4% for noradrenaline, 4.8% for dopamine, 4.6% for renin, 4.7% for aldosterone and 3.8% for plasma rennin activity. An automatic gamma counter (type: Cobra II/5010, company: Packard, Ramsey, MN, USA) was used, to count the radioactivity and calculate the results. Measurement of excreted urine and calculation of creatinine clearance was performed hourly, for the first 8 h, and daily thereafter. Glomerular filtration rate was calculated using the Modification of Diet in Renal Disease (MDRD) equation [14].

### 2.3. Study Endpoints

Primary outcomes of the clinical trial were biochemical and neurohormonal indices including hemoglobin (Hb), electrolytes, BNP, urea, creatinine clearance, dopamine, renin, angiotensin, aldosterone, adrenaline and noradrenaline upon emergency department admission, at 8 and/or 24 h and, thereafter, once daily. 

Secondary outcomes included plasma dopamine levels of patients and their correlation with clinical outcomes including length of stay (LoS), in-hospital mortality, 2-month mortality and rehospitalization, and 1-year mortality and rehospitalization. 

### 2.4. Statistical Analysis

Continuous variables exhibiting normal distribution were presented as means and standard deviations (SD), whereas continuous variables exhibiting non-normal distribution were presented as medians and 25th–75th percentiles. The normality of the distribution of a continuous variable or lack thereof was examined with the Kolmogorov–Smirnov test. Absolute (N) and relative (%) frequencies were used to describe qualitative variables. Pearson’s χ^2^ test or Fisher’s exact test were used to compare proportions. A parametric test of analysis of variance (ANOVA) or the non-parametric Kruskal–Wallis test were used for the comparison of quantitative variables between more than two groups. To check for type I error due to multiple comparisons, the Bonferroni correction was used, whereby the significance level was 0.05/κ (κ = number of comparisons). The non-parametric Wilcoxon-signed rank test was used to compare the values between the different time points. Repeated-measures analysis of variance (ANOVA) was used to test for differences in heart rate, systolic blood pressure (SBP), and diastolic blood pressure (DBP) measurements between groups and different time points. Moreover, the ANOVA assessed whether the degree of change over time of the parameters under study was different between the two groups. Due to the asymmetry of the distributions, logarithmic transformations of the variables in the repeated-measures ANOVA method were used. Deviations from sphericity were assessed using Mauchly’s test. Significance levels were two-sided and statistical significance was set at 0.05. The statistical program SPSS 22.0 was used for the analysis.

## 3. Results

### 3.1. Characteristics of Patients

The total sample consisted of 62 patients (64.5% women) with a mean age of 75 years (SD = 10.1 years), divided into three groups: i. Group 1 (LDF: low dose furosemide) consisting of patients who received low-dose furosemide (N = 23, 37.1%), ii. Group 2 (HDF: high-dose furosemide) consisting of patients who received high-dose furosemide (N = 13, 21%) and iii. Group 3 (LDFD: low-dose furosemide and dopamine) consisting of those who received a combination of low-dose furosemide and dopamine (N = 26, 41.9%). Table 1 demonstrates baseline demographic characteristics and medication of patients in each group.

### 3.2. Outcomes

The effects between different doses of furosemide or combination of furosemide and dopamine on biochemical and neurohormonal indices including hemoglobin (Hb), electrolytes, BNP, urea, creatinine, dopamine, renin, angiotensin, aldosterone, adrenaline and noradrenaline are demonstrated comprehensively in Table 2 and Table 3. 

Hemoglobin, electrolytes, BNP and troponin were similar both at admission and at 24 h among the three groups (*p* > 0.05). Comparing values at hospital admission and after 8 or 24 h within each group, we found that in the LDF group hemoglobin and potassium were significantly reduced at 24 h (*p* < 0.01) and in the HDF group potassium was significantly reduced at 24 h (*p* = 0.013), while in the LDFD group hemoglobin was significantly reduced at 24 h (*p* = 0.001) but troponin was increased at 8 h (*p* < 0.05). 

Noradrenaline and dopamine values at admission were found to be significantly different among the three groups. Specifically, after Bonferroni correction, it was found that noradrenaline values at admission were significantly lower in the HDF group compared to the LDFD group (*p* = 0.020), while dopamine values at admission were significantly lower in the LDF group compared to the LDFD group (*p* = 0.002). Fetuin and dopamine values at 8 h were also found to be significantly different among the three groups. Fetuin values at 8 h were significantly lower in the HDF group compared to the LDFD group (*p* = 0.024), while dopamine values at 8 h were significantly lower in the HDF and LDF groups compared with the LDFD group (*p* < 0.001 for both comparisons) after Bonferroni correction. Comparing these indices within each group between admission and at 8 h, we found that in the HDF group aldosterone, noradrenaline and fetuin significantly decreased at 8 h (*p* = 0.011, *p* = 0.009 and *p* = 0.012, respectively), in the LDFD group aldosterone, adrenaline and fetuin decreased at 8 h (*p* = 0.032, *p* = 0.004 and *p* = 0.08, respectively), while dopamine values increased (*p* < 0.004), and in the LDF group aldosterone and fetuin decreased at 8 h (*p* = 0.003 and *p* = 0.006, respectively).

**Figure 1:**
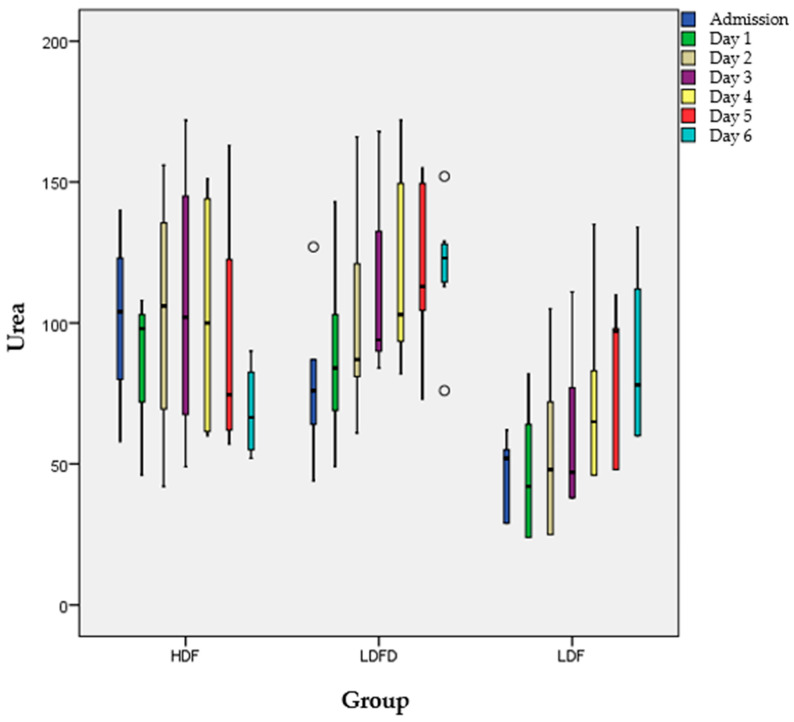
Urea levels from admission to day 6. Circles = Outliers.

**Figure 2:**
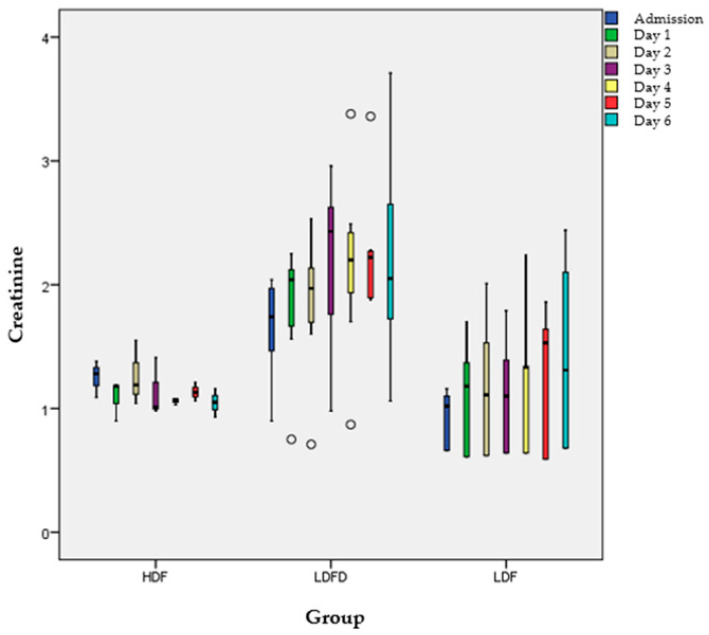
Creatinine levels from admission to day 6. Circles = Outliers.

The amount of urine was similar among the three groups as per measurement at 2, 4, 6 and at 8 h (*p* > 0.05). Moreover, changes in urea levels were also similar among the three groups as per measurement at 2, 4, 6 and at 8 h, as well as the 1-, 2- and 3-day production and balance of urine (*p* > 0.05). Creatinine and urea levels from admission until day 6 are demonstrated in Figure 1 and Figure 2 and Table 3, for each group.

Urea and creatinine levels were similar in each day among the three groups, as well as the number of days until the peak values. Comparing the peak values with the values during admission and at day 1 within each group, we found that in all groups the maximum values of urea and creatinine were significantly different compared to those at admission (*p* < 0.05) and at day 1 (*p* < 0.05), while values on admission and at day were similar (*p* > 0.05).

Clinical outcomes did not differ among the three groups, and are presented in Table 4. Length of stay, in-hospital mortality, 2-month mortality and rehospitalization, and 1-year mortality and rehospitalization did not differ among patients receiving different doses of furosemide, nor in patients receiving furosemide in combination with dopamine.

## 4. Discussion

This secondary analysis of the DAD-HF II [13] trial investigated the neurohormonal effects of the combination of low-dose furosemide and dopamine versus low- or high-dose furosemide only, in patients hospitalized for acute decompensation of HF, as well as the clinical outcomes, including length of hospitalization, in-hospital mortality, 2-month mortality, 2-month hospitalizations, 1-year mortality and 1-year hospitalizations. The present analysis demonstrated that the addition of low-dose (renal-dose) dopamine to intravenous furosemide therapy was not associated with favorable neurohormonal effects or clinical outcomes.

Patients hospitalized for acute decompensated HF may present with prolonged hospital stay, higher in-hospital mortality, increased likelihood of readmission, and increased mortality after discharge [2,3] and WRF, due to high doses of IV furosemide, which is considered as a first-line therapy in acute HF [15,16,17]. The median duration of hospitalization was 5 [3,4,5,6,7,8] days in the LDF, 6 [4,5,6,7,8,9] days in the HDF and 5 [4,5,6,7,8,9,10] days in the LDFD group, without significant differences between the three groups (*p* = 0.523). The median duration of ICU stay was 3 [2,3,4,5] days in the LDF, 4 [3,4,5,6] days in the HFD and 3 [2,3,4,5] days in the LDFD group. Similarly, there were no significant differences between the three groups (*p* = 0.4995). Heart failure (HF) and hospitalization length-of-stay (LOS), especially in the Intensive Care Unit (ICU), have been associated with the risk of subsequent readmission and mortality [18,19]. In the current analysis, the lack of significant differences in biochemical and neurohormonal indices was translated to similar length of total hospitalization and ICU hospitalization among the three groups. 

The normal ceiling daily dose of furosemide is 80 mg once or twice daily, increasing up to 160 and 240 mg in patients with chronic kidney disease (CKD) stages 3 and 4 or nephrotic syndrome, or 80 to 160 mg in patients with cirrhosis or HF with preserved glomerular filtration rate (GFR), while very high doses of 500 mg may be required in patients with end-stage CKD [20,21]. Patients with CHF may require higher doses of furosemide given twice daily, due to the impaired absorption of loop diuretics and impaired tubular response that they may present [22]. A common pathophysiological characteristic of these patients is diuretic resistance, which implies a failure to increase fluid and sodium (Na^+^) output sufficiently to relieve volume overload, edema, or congestion [20]. Diuretic resistance is a major cause of recurrent hospitalizations, and may even predict mortality [20]. Nephrotic syndrome in HF is a major cause of mucosal edema of the intestine, thus limiting the absorption of diuretics [22]. Other causes of diuretic resistance include nonadherence to recommended sodium and/or fluid restriction, low doses or low frequency or poor absorption of diuretics, reduced diuretic secretion due to decreased kidney blood flow or functional kidney mass, and insufficient kidney response to diuretics due to low glomerular filtration rate, decreased effective intravascular volume despite elevated total extracellular fluid volume, activation of the renin–angiotensin system, nephron adaptation, or use of nonsteroidal anti-inflammatory drugs [23,24].

Heart failure is a clinical syndrome characterized by neurohormonal activation [25]. Although the activation of these neurohormonal systems (i.e., renin–angiotensin–aldosterone system, sympathetic nervous system, and natriuretic peptides) preserves cardiovascular homeostasis in the beginning of HF and in the short term, a plethora of pre-clinical and clinical studies have demonstrated that the bioactive molecules generated by the activation of these systems are becoming toxic to the heart at higher levels [26]. Renal failure presents an obstacle, and is a major challenge in managing HF patients. HF can compromise renal function due to impaired cardiac output and kidney perfusion, while HF medications may cause renal function to deteriorate. The present work demonstrated that the addition of low-dose dopamine in acute HF patients treated with iv furosemide was not associated with beneficial renoprotective (i.e., urea, creatinine, worsening renal-function events) and neurohormonal (i.e., adrenaline, noradrenaline, renin, angiotensin, aldosterone, troponin, and natriuretic peptides) effects. Urea is an established predictor of all-cause mortality in HF patients. A recent meta-analysis of 19 cohort studies involving 56,003 patients revealed a strong association between urea values and mortality in HF patients [27]. In particular, when blood urea nitrogen (BUN) was used as a categorical variable, the risk of death from heart failure was 2.29 times higher for high levels of BUN than for low levels of BUN (RR = 2.29, 95% CI:1.42–3.70, *p* < 0.001), whereas when BUN was used as a continuous variable, the risk of death from HF was 1.02 times higher for each unit increase in BUN (RR = 1.02, 95% CI:1.01–1.03, *p* < 0.001) [27]. However, worsening renal function (i.e., a serum creatinine level increase of ≥0.3 mg/dL) is not an independent determinant of outcomes in acute-HF patients [28]. In the present study, both urea and creatinine were not significantly different between the three groups the first 6 days of hospitalization. The decrease in fetuin observed in the three groups may reflect the impaired systemic anti-inflammatory activity [29] in acute-HF patients.

A variety of vasoactive drugs have been tested over the years in order to preserve renal function in these patients, including nesiritide [30,31], nitroglycerin [32], nitroprusside [33], and dobutamine [34], but they all either failed or did not manage to demonstrate a stable beneficial effect, except for the increase in cardiac output. Only low-dose dopamine was demonstrated to increase renal blood flow and GFR in stable patients with systolic HF, thus enhancing decongestion and preserving renal function [7]. Dopamine at renal doses of 0.5–5 mg per kg per min presents mild positive inotropic- and afterload-reducing effects, stimulates the renal dopaminergic receptors, and promotes a decrease in renal vascular resistance, an increase in renal blood flow, and an increase in GFR [6]. Apart from this, a characteristic of dopamine is also the direct natriuretic and diuretic effect mediated by the stimulation of the dopamine alpha-1 and dopamine alpha-2 receptors in the proximal tubule, the thick ascending loop of Henle, and the cortical collecting ducts [35]. Indeed, a large meta-analysis of 61 trials that randomly assigned 3359 patients at risk of acute renal failure to receive low-dose dopamine (5 mg per kg per min) or placebo, showed the renoprotective effects of dopamine and its association, with an improvement in renal physiology 24 h after initiation of infusion by a 24% increase in the urine output, a 4% relative decrease in serum creatinine level, and a 6% relative increase in the measured creatinine clearance [36]. On the contrary, similarly to the present work, there were nonrandomized controlled trials that did not manage to show beneficial renoprotective effects of dopamine [37,38,39]. Interestingly, dopamine administration in this study was not associated with augmented urine output or reduced natriuretic peptides at 8 h after admission. 

The DAD-HF trial [10] showed that the combination of low-dose furosemide and low-dose dopamine is equally effective as high-dose furosemide, but is associated with improved renal function profile as expressed by the number of patients with WRF [more frequent in the HDF (30%) than in the LDFD group (6.7%); *p* = 0.042] and improved potassium homeostasis [from 4.36 ± 0.5 to 3.96 ± 0.4 mEq/L at 24 h, *p* = 0.003) in the HDF group and from 4.46 ± 0.5 to 4.26 ± 0.5 mEq/L at 24 h, *p* = 0.07 in the LDFD group; *p* = 0.027 between groups]. The addition of low-dose dopamine may have contributed to an amelioration of the furosemide-induced kidney injury. Other clinical trials performed in the following years showed beneficial effects of dopamine on renal function, diuresis and symptom relief, without a significant increase in troponin-T in acute-HF patients with diuretic resistance [11], or improved clinical outcomes in HF with reduced ejection fraction, as shown in a post hoc analysis of the ROSE AHF trial [40]. Moreover, a meta-analysis by Xing F et al. [41], examined the effects of low-dose dopamine on blood urea, creatinine levels, eGFR and urine output in 587 HF patients, indicating benefits in diuresis and preservation of renal function. However, the same meta-analysis did not show the superiority of dopamine on all-cause mortality and readmission after treatment in these patients [41]. The most recent clinical study performed by Yang L et al. [12] assessed the impact of tolvaptan and low-dose dopamine on HF patients with acute kidney injury by randomizing them for either receiving tolvaptan 15 mg orally daily for a week or receiving combination treatment of oral tolvaptan 15 mg and dopamine infusion (2 μg per kg per min) for the same time period. Patients receiving the combined therapy demonstrated lower levels of indexes related to cardiac function, including NT-proBNP and cardiac troponin I, as well as lower levels of renal function, including serum cystatin C, serum creatinine, and neutrophil gelatinase-associated lipocalin, indicating that the addition of low-dose dopamine may improve cardiac and renal function in HF.

The DAD-HF II trial [13] was a single, blind, randomized controlled trial, including 161 patients with acute decompensated HF. Patients were randomized to 8 h continuous infusions of high-dose furosemide of 20 mg/h (HDF group), low-dose furosemide and low-dose dopamine of 5 mg/h and 5 μg per kg per min, respectively (LDFD group), or low-dose furosemide of 5 mg/h (LDF group). The 60-day all-cause mortality (4.0%, 7.1%, and 7.2% in HDF, LDFD, and LDF groups, respectively; *p* = 0.74), one-year all-cause mortality (38.1%, 33.9% and 32.7% in HDF, LDFD, and LDF groups, respectively; *p* = 0.84) and hospitalization for HF [60-day: 22.0%, 21.4%, and 14.5% in HDF, LDFD, and LDF groups, respectively (*p* = 0.55); 1-year: 60.0%, 50.0%, and 47% (*p* = 0.40)], as well as other indices such as dyspnea, WRF, and length of stay, showed no significant differences between high- versus low-dose furosemide infusion groups. Moreover, the addition of low-dose dopamine infusion was not associated with any beneficial effects in the above indices. Similar findings were demonstrated the same year by another group of investigators in the United States and Canada. Chen HH et al. performed a multicenter, double-blind, placebo-controlled randomized clinical trial (The ROSE Acute Heart Failure Randomized Trial) [42]. The authors randomized 360 hospitalized participants with acute HF and renal dysfunction (estimated GFR of 15–60 mL/min/1.73m^2^) in either a low-dose dopamine group of 2 μg/kg/min or a low-dose nesiritide group of 0.005 μg/kg/min, added to diuretic therapy, and compared them to the placebo in order to evaluate the decongestion endpoint assessed by the 72 h cumulative urine volume and the renal function endpoint assessed by the 72 h change in serum cystatin-C. The authors concluded that neither low-dose dopamine nor low-dose nesiritide showed significant effect on 72 h cumulative urine volume or on the change in cystatin-C, compared to the placebo. Indeed, in our study we did not manage to find any significant differences for neurohormonal effects and clinical outcomes between different doses of furosemide. Moreover, the addition of low-dose dopamine to the furosemide therapy also did not show any acute beneficial effects after 8 or 24 h of admission, or for clinical outcomes after 2 months and/or 1 year. Another recent randomized, single-blinded study assessing the effects of low-dose dopamine on renal function in patients with HF, the ROPA-DOP trial, was conducted in the U.S. [43]. Investigators randomized 90 hospitalized patients with acute HF with preserved ejection fraction within 24 h of admission for either furosemide treatment only or for furosemide treatment with low-dose dopamine, and assessed creatinine from baseline to 72 h. The authors did not observe beneficial effects of low-dose dopamine on renal function in these patients (low-dose dopamine: 12.79%; 95% CI: 5.66% to 19.92%, vs. no-dopamine: 8.03%; 95% CI: 1.44% to 14.62%; *p* = 0.33), while a continuous infusion diuretic strategy was associated with renal impairment, compared to intermittent bolus (odds ratio:4.32; 95% CI: 1.26 to 14.74; *p* = 0.02).

Our study has some limitations. Initially, our sample size was not large, including only 62 patients hospitalized for acute decompensation of HF from our center (Larissa University Hospital) and thus, our results may not be safely generalizable for this population. However, the present work was a secondary analysis of the randomized DAD-HF II trial. Moreover, due to the small sample size, some of our comparisons might be underpowered, and this may be the reason that we did not observe statistically significant differences among groups. Another limitation of the current analysis is the lack of adjustment for potential confounding variables, such as baseline differences or comorbidities among patient groups. However, post hoc randomized analyses generally have superior statistical validity because they inherently control for both known and unknown confounders, through randomization [44].

## 5. Conclusions

The addition of low-dose dopamine to intravenous furosemide therapy in patients with diuretic resistance hospitalized for acute decompensation of HF was not associated with significant differences with respect to neurohormonal effects and clinical outcomes. Whether the addition of low-dose dopamine to intravenous furosemide proves to be beneficial in distinct acute HF phenotypes needs to be evaluated in studies with a larger sample size. 

## Figures and Tables

**Table 1:** Baseline characteristics of patients of each group.

Demographics	Groups			*p* Value between Groups
	Group 1 (LDF) N = 23	Group 2 (HDF) N = 13	Group 3 (LDFD) N = 26	
	N (%)	N (%)	N (%)	
Age (years) ^a^	75.2 ± 10.1	78 ± 5.9	73.2 ± 11.7	0.386 ^‡^
Systolic blood pressure (mmHg)	180 (165–190)	172 (156–200)	184 (170–210)	0.554 ^#^
Diastolic blood pressure (mmHg)	87 (74–100)	76 (70–102)	90.5 (84–100)	0.302 ^#^
Heart rate (bpm)	92 (83–108)	91 (74–100)	92 (83–108)	0.028 ^#^
Gender (females)	17 (73.9)	8 (61.5)	15 (57.7)	0.480 ^+^
ΒΜΙ (>30 kg/m^2^) ^a^	28.7 ± 5.9	30.4 ± 2.0	27.4 ± 4.2	0.168 ^‡^
Previous medical history				
Heart failure (%)	14 (60.9)	7 (53.8)	11 (42.3)	0.424 ^+^
Diabetes mellitus (%)	16 (69.6)	8 (61.5)	11 (42.3)	0.145 ^+^
Hypertension (%)	23 (100)	12 (92.3)	25 (96.2)	0.684 ^+ +^
Dyslipidemia (%)	13 (56.5)	6 (46.2)	15 (57.7)	0.776 ^+^
CAD (%)	9 (39.1)	5 (38.5)	13 (50.0)	0.684 ^+^
Atrial fibrillation (%)	11 (47.8)	1 (7.7)	7 (26.9)	0.037 ^+^
Hematology tests				
Hemoglobin (g/dL)	12.7 (11–14.6)	12.2 (11.4–12.6)	12 (10.9–14.3)	0.754 ^#^
Urea (mg/dL)	49 (35–70)	69 (49–96)	49.5 (43–76)	0.109 ^#^
Creatinine (mg/dL)	1.1 (0.8–1.5)	1.3 (1.2–1.4)	1.3 (1.1–1.8)	0.058 ^#^
Medication				
b-blockers (%)	12 (52.2)	10 (76.9)	13 (50.0)	0.243 ^+^
ACEi/ARB (%)	14 (60.9)	8 (61.5)	16 (61.5)	0.999 ^+^
MRAs (%)	3 (13%)	2 (15.4)	3 (11.5)	1.000 ^+ +^
Diuretics (%)	14 (60.9)	10 (76.9)	17 (65.4)	0.617 ^+^
Echocardiography indices				
EF ^b^ (%)	35 (25–45)	35 (20–45)	30 (25–45)	0.787 ^#^
LVEDD ^b^ (mm)	59.8 (54–62)	56 (54.5–56)	57 (50–61.5)	0.911 ^#^

LDF, low-dose furosemide; HDF, high-dose furosemide; LDFD, low-dose furosemide and dopamine; BMI, body mass index; CAD, coronary artery disease; ACEi, angiotensin-converting enzyme inhibitors; ARB, angiotensin receptor blockers; bpm, beats per minute; EF, ejection fraction; LVEDD, left ventricular end-diastolic diameter; MRA, mineralocorticoid receptor antagonist. ^a^ mean ± standard deviation ^b^ median (25th–75th percentiles). ^+^ Pearson’s χ^2^ test ^+ +^ Fisher’s exact test ^‡^ ANOVA ^#^ Kruskal–Wallis test.

**Table 2:** Effects of different doses of furosemide or combination of furosemide and dopamine on biochemical and neurohormonal indices between different time points.

Biochemical and Neurohormonal Indices ^a^	Groups			*p* Value between Groups ^‡^
	Group 1 (LDF) N = 23	Group 2 (HDF) N = 13	Group 3 (LDFD) N = 26	
Hb (g/dL)				
ED admission	12.7 (11–14.6)	12.2 (11.4–12.6)	12 (10.9–14.3)	0.754
24 h	10.8 (9.5–13) *	10.4 (9.6–12.5)	11.1 (10.4–12.2) *	0.906
Na (mEq/L)				
ED admission	138 (135–139)	137 (132–140)	137 (133–140)	0.883
24 h	137 (136–139)	138 (136–139)	136.5 (134–140)	0.858
K (mEq/L)				
ED admission	4.4 (4.2–4.8)	4.3 (3.9–5.3)	4.2 (3.9–4.6)	0.196
24 h	4.1 (3.5–4.4) *	3.8 (3.5–4.5) *	3.9 (3.8–4.4)	0.824
BNP (pg/mL)				
ED admission	544.5 (352–881)	803 (525–1760)	702.5 (486.5–1195)	0.080
8 h	871 (556–1170)	790 (614–1431)	654 (392–1310)	0.783
Troponin (ng/mL)				
ED admission	0.1 (0.1–0.1)	0.1 (0–0.2)	0.1 (0–0.1)	0.696
8 h	0.1 (0.1–0.1)	0.1 (0.1–0.1)	0.1 (0.1–0.3) *	0.959
Renin (μIU/mL)				
ED admission	25 (5.7–58.8)	6.3 (3.3–20.6)	10.8 (6.2–30.3)	0.242
8 h	17.3 (5.1–57.2)	7 (3.3–24.7)	12.7 (6.4–22.9)	0.312
Angiotensin (ng/mL)				
ED admission	1.7 (0.2–6.1)	0.7 (0.2–3.7)	0.9 (0.4–3.4)	0.674
8 h	1.2 (0.3–3.9)	0.6 (0.4–3)	0.6 (0.3–1.8)	0.683
Aldosterone (pg/mL)				
ED admission	102.3 (43.7–599.4)	202.1 (113.1–547.4)	191.4 (130–317.1)	0.337
8 h	62.2 (28.9–144.5) *	114.2 (64.1–222.6) *	106.8 (59.5–175.1) *	0.153
Adrenaline (ng/L)				
ED admission	56.3 (26.3–85)	40 (32–83.1)	112.5 (40.9–228.4)	0.162
8 h	39.1 (22–84.1)	26.4 (21.7–51.9)	46.3 (23.5–67.7) *	0.625
Noradrenaline (ng/L)				
ED admission	453.9 (295.3–1803)	385.4 (258.6–760)	1029.2 (505.1–1836)	0.050
8 h	545.7 (296.6–924.4)	315.5 (55–450.6) *	438.4 (308.1–1517)	0.104
Dopamine (ng/L)				
ED admission	58.9 (21.1–167.6)	77.5 (48.6–126)	174.3 (80.1–1380)	0.005
8 h	59.8 (25.3–89)	61.9 (14.6–116.2)	5542 (482.3–13,737.5) *	<0.001
Fetuin (g/L)				
ED admission	0.3 (0.2–0.3)	0.2 (0.2–0.3)	0.3 (0.2–0.3)	0.159
8 h	0.2 (0.2–0.2) *	0.2 (0.1–0.2) *	0.2 (0.2–0.3) *	0.043

LDF, low-dose furosemide; HDF, high-dose furosemide; LDFD, low-dose furosemide and dopamine; ED, emergency department; Hb, hemoglobin; Na, sodium; K, potassium; BNP, brain natriuretic peptide. ^a^ Median (25th–75th percentiles). ^‡^ Kruskal–Wallis test. * *p* < 0.05 between the 2 time points within each group.

**Table 3:** Creatinine and urea levels from admission until day 6 and other renal indices between different time points.

Time Point	Groups						*p* Value for Urea Levels between Groups ^‡^	*p* Value for Creatinine Levels between Groups ^‡^
	Group 1 (LDF) N = 23		Group 2 (HDF) N = 13		Group 3 (LDFD) N = 26			
	Urea ^a^ (mg/dL)	Creatinine ^a^ (mg/dL)	Urea ^a^ (mg/dL)	Creatinine ^a^ (mg/dL)	Urea ^a^ (mg/dL)	Creatinine ^a^ (mg/dL)		
ED admission	49 (35–70)	1.1 (0.8–1.5)	69 (49–96)	1.3 (1.2–1.4)	49.5 (43–76)	1.3 (1.1–1.8)	0.109	0.058
Day 1	53 (35–82)	1.2 (0.9–1.6)	63 (52–98)	1.3 (1.2–1.4)	51.5 (39–84)	1.2 (1–1.8)	0.284	0.340
Day 2	57 (43–82)	1.2 (0.8–1.5)	78 (61–97)	1.3 (1.2–1.6)	62 (44–103)	1.3 (1–2)	0.319	0.332
Day 3	74 (52–105.5)	1.2 (0.9–1.4)	68 (53–106)	1.2 (1–1.6)	87.5 (53–100)	1.5 (1.1–2.4)	0.908	0.183
Day 4	71 (65–106)	1.3 (0.9–1.6)	77 (63–103)	1.2 (1.1–1.5)	97 (70–125)	1.7 (1.3–2.2)	0.699	0.216
Day 5	94 (59–110)	1.5 (1–1.6)	82 (67–102)	1.2 (1.1–1.8)	111 (73–153)	1.9 (1.3–2.3)	0.636	0.268
Day 6	78 (60–134)	1.3 (0.7–2.4)	75 (58–90)	1.1 (1–1.7)	125 (114.5–140.5)	2.1 (1.7–2.9)	0.192	0.144
Peak value	81.5 (70–113) *^+^	1.4 (1–2.1) *^+^	102 (70–163) *^+^	1.5 (1.3–1.9) *^+^	103 (65–127) *^+^	1.5 (1.2–2.8) *^+^	0.812	0.267
Days until peak value	4 (3–5)	3 (1–5)	3.5 (2–5)	2 (1–4)	4 (3–5)	3 (2–4)	0.894	0.809
Urine output at 2 h (mL)	1320 (700–1700)		970 (850–1150)		1035 (570–1500)		0.678 ^‡^	
Urine output at 4 h (mL)	1840 (1200–2280)		1390 (1270–1780)		1330 (720–2000)		0.316 ^‡^	
Urine output at 6 h (mL)	2430 (1650–2740)		1860 (1670–2250)		1615 (1160–2720)		0.199 ^‡^	
Urine output at 8 h (mL)	2890 (2000–3210)		2330 (2110–2600)		2020 (1440–2960)		0.187 ^‡^	
WRFcr at day 1 (mg/dL), Ν (%)	2 (8.7)		0 (0)		3 (11.5)		0.715 ^¶¶^	
WRFcr peak value (mg/dL), Ν (%)	10 (52.6)		5 (50.0)		12 (54.5)		0.971 ^¶^	

LDF, low-dose furosemide; HDF, high-dose furosemide; LDFD, low-dose furosemide and dopamine; ED, emergency department; WRFcr, worsening renal function—creatinine. WRFcr is defined as an increase in serum creatinine levels ≥ 0.3 mg/dL. ^a^ Median (25th–75th percentiles). ^‡^ Kruskal–Wallis test; ^¶^ Pearson’s χ^2^ test; ^¶¶^ Fisher’s exact test. * *p* < 0.05 compared to admission within each group + *p* < 0.05 compared to day 1 within each group.

**Table 4:** Clinical outcomes of patients of each group.

Clinical Outcome	Groups			*p* Value between Groups
	Group 1 (LDF) N = 23	Group 2 (HDF) N = 13	Group 3 (LDFD) N = 26	
	N (%)	N (%)	N (%)	
Length of stay (days) ^a^	5 (3–8)	6 (4–9)	5 (4–10)	0.523 ^‡^
In-hospital mortality	1 (4.3)	0 (0)	1 (3.8)	1.000 ^+ +^
2-month mortality	2 (9.1)	0 (0)	3 (12.5)	0.168 ^‡^
2-month rehospitalization	3 (14.3)	1 (8.3)	5 (21.7)	0.716 ^+ +^
1-year mortality	6 (27.3)	3 (37.5)	7 (35.0)	0.811 ^+^
1-year rehospitalization	9 (47.4)	6 (60.0)	8 (47.1)	0.774 ^+^

LDF, low-dose furosemide; HDF, high-dose furosemide; LDFD, low-dose furosemide and dopamine. ^a^ Median (25th–75th percentiles). ^+^ Pearson’s χ^2^ test; ^+ +^ Fisher’s exact test; ^‡^ Kruskal–Wallis test.

## Data Availability

Data available upon request.

## References

[1] Mullens W., Abrahams Z., Francis G.S., Sokos G., Taylor D.O., Starling R.C., Young J.B., Tang W.H.W. (2009). Importance of venous congestion for worsening of renal function in advanced decompensated heart failure. J. Am. Coll. Cardiol..

[2] Akhter M.W., Aronson D., Bitar F., Khan S., Singh H., Singh R.P., Burger A.J., Elkayam U. (2004). Effect of elevated admission serum creatinine and its worsening on outcome in hospitalized patients with decompensated heart failure. Am. J. Cardiol..

[3] Giamouzis G., Kalogeropoulos A.P., Georgiopoulou V.V., Agha S.A., Rashad M.A., Laskar S.R., Smith A.L., Butler J. (2009). Incremental value of renal function in risk prediction with the Seattle Heart Failure Model. Am. Heart J..

[4] Felker G.M., O’Connor C.M., Braunwald E., Heart Failure Clinical Research Network Investigators (2009). Loop diuretics in acute decompensated heart failure: Necessary? Evil? A necessary evil?. Circ. Heart Fail..

[5] Bozkurt B., Kamat I.S. (2019). Worsening Renal Function in Acute Decompensated Heart Failure: A Bad Sign, or Maybe Not?. Trans. Am. Clin. Clim. Assoc..

[6] Elkayam U., Ng T.M., Hatamizadeh P., Janmohamed M., Mehra A. (2008). Renal Vasodilatory Action of Dopamine in Patients with Heart Failure: Magnitude of Effect and Site of Action. Circulation.

[7] Bistola V., Arfaras-Melainis A., Polyzogopoulou E., Ikonomidis I., Parissis J. (2019). Inotropes in Acute Heart Failure: From Guidelines to Practical Use: Therapeutic Options and Clinical Practice. Card. Fail. Rev..

[8] Ho D., Yan L., Iwatsubo K., Vatner D.E., Vatner S.F. (2010). Modulation of beta-adrenergic receptor signaling in heart failure and longevity: Targeting adenylyl cyclase type 5. Heart Fail. Rev..

[9] Kindgen-Milles D., Tarnow J. (1997). Low dosage dopamine improves kidney function: Current status of knowledge and evaluation of a controversial topic. Anasthesiol. Intensiv. Notfallmed Schmerzther..

[10] Giamouzis G., Butler J., Starling R.C., Karayannis G., Nastas J., Parisis C., Rovithis D., Economou D., Savvatis K., Kirlidis T. (2010). Impact of dopamine infusion on renal function in hospitalized heart failure patients: Results of the Dopamine in Acute Decompensated Heart Failure (DAD-HF) Trial. J. Card. Fail..

[11] Kamiya M., Sato N., Nozaki A., Akiya M., Okazaki H., Takahashi Y., Mizuno K., Shimizu W. (2015). Renal effects of added low-dose dopamine in acute heart failure patients with diuretic resistance to natriuretic peptide. J. Cardiovasc. Pharmacol..

[12] Yang L., Wang J., Yu Y., Li Y., Zhang S. (2024). Impact of Tolvaptan Combined with Low-Dose Dopamine in Heart Failure Patients with Acute Kidney Injury. Int. Heart J..

[13] Triposkiadis F.K., Butler J., Karayannis G., Starling R.C., Filippatos G., Wolski K., Parissis J., Parisis C., Rovithis D., Koutrakis K. (2014). Efficacy and safety of high dose versus low dose furosemide with or without dopamine infusion: The Dopamine in Acute Decompensated Heart Failure II (DAD-HF II) trial. Int. J. Cardiol..

[14] Modification of Diet in Renal Disease Study Group (1992). The Modification of Diet in Renal Disease Study: Design, methods, and results from the feasibility study. Am. J. Kidney Dis..

[15] McDonagh T.A., Metra M., Adamo M., Gardner R.S., Baumbach A., Bohm M., Burri H., Butler J., Celutkiene J., Chioncel O. (2021). 2021 ESC Guidelines for the diagnosis and treatment of acute and chronic heart failure. Eur. Heart J..

[16] McDonagh T.A., Metra M., Adamo M., Gardner R.S., Baumbach A., Bohm M., Burri H., Butler J., Celutkiene J., Chioncel O. (2023). 2023 Focused Update of the 2021 ESC Guidelines for the diagnosis and treatment of acute and chronic heart failure. Eur. Heart J..

[17] Heidenreich P.A., Bozkurt B., Aguilar D., Allen L.A., Byun J.J., Colvin M.M., Deswal A., Drazner M.H., Dunlay S.M., Evers L.R. (2022). 2022 AHA/ACC/HFSA Guideline for the Management of Heart Failure: A Report of the American College of Cardiology/American Heart Association Joint Committee on Clinical Practice Guidelines. Circulation.

[18] Reynolds K., Butler M.G., Kimes T.M., Rosales A.G., Chan W., Nichols G.A. (2015). Relation of Acute Heart Failure Hospital Length of Stay to Subsequent Readmission and All-Cause Mortality. Am. J. Cardiol..

[19] Cotter G., Davison B.A., Milo O., Bourge R.C., Cleland J.G., Jondeau G., Krum H., O’Connor C.M., Metra M., Parker J.D. (2016). Predictors and Associations with Outcomes of Length of Hospital Stay in Patients with Acute Heart Failure: Results from VERITAS. J. Card. Fail..

[20] Wilcox C.S., Testani J.M., Pitt B. (2020). Pathophysiology of Diuretic Resistance and Its Implications for the Management of Chronic Heart Failure. Hypertension.

[21] Hoorn E.J., Wilcox C.S., Ellison D.H. (2020). Chapter 50, diuretics. Brenner and Rector’s the Kidney.

[22] Brater D.C. (1994). Pharmacokinetics of loop diuretics in congestive heart failure. Br. Heart J..

[23] Hoorn E.J., Ellison D.H. (2017). Diuretic Resistance. Am. J. Kidney Dis..

[24] Hoorn E.J., Wilcox C.S., Ellison D.H., Skorecki K., Chertow G., Marsden P., Taal M., Yu A. (2015). Diuretics. Brenner and Rector’s the Kidney.

[25] Xanthopoulos A., Skoularigis J., Triposkiadis F. (2023). The Neurohormonal Overactivity Syndrome in Heart Failure. Life.

[26] Manolis A.A., Manolis T.A., Manolis A.S. (2023). Neurohumoral Activation in Heart Failure. Int. J. Mol. Sci..

[27] Duan S., Li Y., Yang P. (2023). Predictive value of blood urea nitrogen in heart failure: A systematic review and meta-analysis. Front. Cardiovasc. Med..

[28] Metra M., Davison B., Bettari L., Sun H., Edwards C., Lazzarini V., Piovanelli B., Carubelli V., Bugatti S., Lombardi C. (2012). Is worsening renal function an ominous prognostic sign in patients with acute heart failure? The role of congestion and its interaction with renal function. Circ. Heart Fail..

[29] Chekol Abebe E., Tilahun Muche Z., Behaile T/Mariam A., Mengie Ayele T., Mekonnen Agidew M., Teshome Azezew M., Abebe Zewde E., Asmamaw Dejenie T., Asmamaw Mengstie M. (2022). The structure, biosynthesis, and biological roles of fetuin-A: A review. Front. Cell Dev. Biol..

[30] Owan T.E., Chen H.H., Frantz R.P., Karon B.L., Miller W.L., Rodeheffer R.J., Hodge D.O., Burnett J.C., Redfield M.M. (2008). The effects of nesiritide on renal function and diuretic responsiveness in acutely decompensated heart failure patients with renal dysfunction. J. Card. Fail..

[31] Wang D.J., Dowling T.C., Meadows D., Ayala T., Marshall J., Minshall S., Greenberg N., Thattassery E., Fisher M.L., Rao K. (2004). Nesiritide does not improve renal function in patients with chronic heart failure and worsening serum creatinine. Circulation.

[32] Elkayam U., Bitar F., Akhter M.W., Khan S., Patrus S., Derakhshani M. (2004). Intravenous nitroglycerin in the treatment of decompensated heart failure: Potential benefits and limitations. J. Cardiovasc. Pharmacol. Ther..

[33] Leier C.V., Bambach D., Thompson M.J., Cattaneo S.M., Goldberg R.J., Unverferth D.V. (1981). Central and regional hemodynamic effects of intravenous isosorbide dinitrate, nitroglycerin and nitroprusside in patients with congestive heart failure. Am. J. Cardiol..

[34] Bayram M., De Luca L., Massie M.B., Gheorghiade M. (2005). Reassessment of dobutamine, dopamine, and milrinone in the management of acute heart failure syndromes. Am. J. Cardiol..

[35] Seri I., Kone B.C., Gullans S.R., Aperia A., Brenner B.M., Ballermann B.J. (1990). Influence of Na+ intake on dopamine-induced inhibition of renal cortical Na(+)-K(+)-ATPase. Am. J. Physiol..

[36] Friedrich J.O., Adhikari N., Herridge M.S., Beyene J. (2005). Meta-analysis: Low-dose dopamine increases urine output but does not prevent renal dysfunction or death. Ann. Intern. Med..

[37] Bellomo R., Chapman M., Finfer S., Hickling K., Myburgh J. (2000). Low-dose dopamine in patients with early renal dysfunction: A placebo-controlled randomised trial. Australian and New Zealand Intensive Care Society (ANZICS) Clinical Trials Group. Lancet.

[38] Lauschke A., Teichgraber U.K., Frei U., Eckardt K.U. (2006). ‘Low-dose’ dopamine worsens renal perfusion in patients with acute renal failure. Kidney Int..

[39] Kellum J.A., Decker J.M. (2001). Use of dopamine in acute renal failure: A meta-analysis. Crit. Care Med..

[40] Wan S.H., Stevens S.R., Borlaug B.A., Anstrom K.J., Deswal A., Felker G.M., Givertz M.M., Bart B.A., Tang W.H., Redfield M.M. (2016). Differential Response to Low-Dose Dopamine or Low-Dose Nesiritide in Acute Heart Failure with Reduced or Preserved Ejection Fraction: Results From the ROSE AHF Trial (Renal Optimization Strategies Evaluation in Acute Heart Failure). Circ. Heart Fail..

[41] Xing F., Hu X., Jiang J., Ma Y., Tang A. (2016). A meta-analysis of low-dose dopamine in heart failure. Int. J. Cardiol..

[42] Chen H.H., Anstrom K.J., Givertz M.M., Stevenson L.W., Semigran M.J., Goldsmith S.R., Bart B.A., Bull D.A., Stehlik J., LeWinter M.M. (2013). Low-dose dopamine or low-dose nesiritide in acute heart failure with renal dysfunction: The ROSE acute heart failure randomized trial. JAMA.

[43] Sharma K., Vaishnav J., Kalathiya R., Hu J.R., Miller J., Shah N., Hill T., Sharp M., Tsao A., Alexander K.M. (2018). Randomized Evaluation of Heart Failure With Preserved Ejection Fraction Patients with Acute Heart Failure and Dopamine: The ROPA-DOP Trial. JACC Heart Fail..

[44] Concato J., Shah N., Horwitz R.I. (2000). Randomized, controlled trials, observational studies, and the hierarchy of research designs. N. Engl. J. Med..

